# Long-ranged Protein-glycan Interactions Stabilize von Willebrand Factor A2 Domain from Mechanical Unfolding

**DOI:** 10.1038/s41598-018-34374-y

**Published:** 2018-10-30

**Authors:** Chuqiao Dong, Jumin Lee, Seonghoon Kim, Whitney Lai, Edmund B. Webb, Alparslan Oztekin, X. Frank Zhang, Wonpil Im

**Affiliations:** 1 Department of Mechanical Engineering and Mechanics, Lehigh University, Bethlehem, PA 18015 United States; 2 Department of Biological Sciences, Lehigh University, Bethlehem, PA 18015 United States; 3 Department of Bioengineering, Lehigh University, Bethlehem, PA 18015 United States

## Abstract

von Willebrand Factor (vWF) is a large multimeric protein that binds to platelets and collagen in blood clotting. vWF A2 domain hosts a proteolytic site for ADAMTS13 (A Disintegrin and Metalloprotease with a ThromboSpondin type 1 motif, member 13) to regulate the size of vWF multimers. This regulation process is highly sensitive to force conditions and protein-glycan interactions as the process occurs in flowing blood. There are two sites on A2 domain (N1515 and N1574) bearing various N-linked glycan structures. In this study, we used molecular dynamics (MD) simulation to study the force-induced unfolding of A2 domain with and without a single N-linked glycan type on each site. The sequential pullout of β-strands was used to represent a characteristic unfolding sequence of A2. This unfolding sequence varied due to protein-glycan interactions. The force-extension and total energy-extension profiles also show differences in magnitude but similar characteristic shapes between the systems with and without glycans. Systems with N-linked glycans encountered higher energy barriers for full unfolding and even for unfolding up to the point of ADAMTS13 cleavage site exposure. Interestingly, there is not much difference observed for A2 domain structure itself with and without glycans from standard MD simulations, suggesting roles of N-glycans in A2 unfolding through long-ranged protein-glycan interactions.

## Introduction

**Figure 1:**
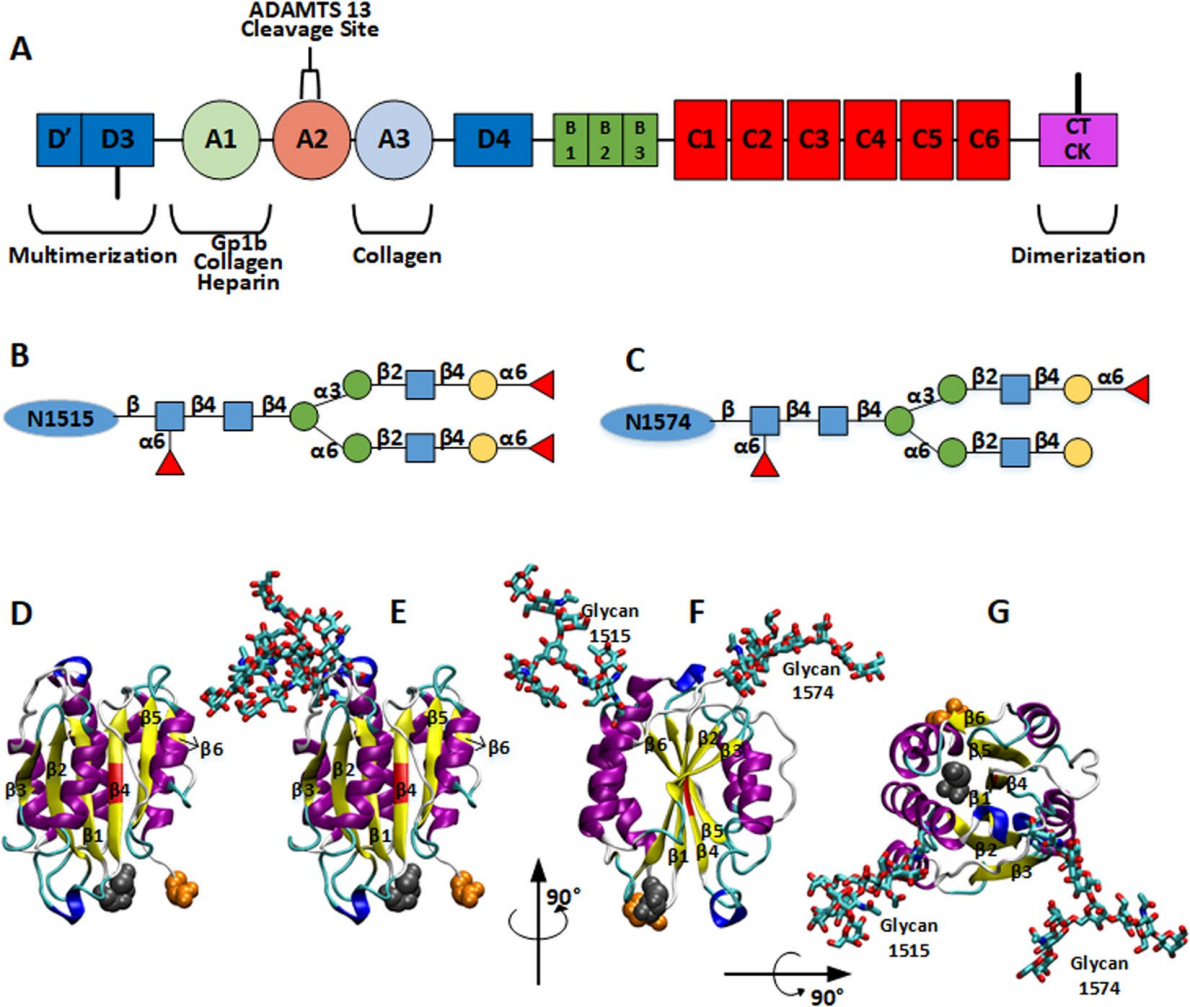
(**A**) Simplified domain sequence of vWF monomer. CK-CK are connected by disulfide bond for dimerization and D3-D3 for multimerization. A2 is the extendable domain and the cleavage site for ADAMTS13 is shielded in its center. (**B**,**C**) Glycan structures used in this work for residues N1515 and N1574, respectively. Specifically, N-acetylglucosamine is represented by blue square, mannose by green circle, galactose by yellow circle, and Fucose by red triangle. (**D**–**G**) Structures for vWF A2 domain with or without glycans at N1515 and N1574. β-strands were labeled according to their positions. (**D**) Structure without glycans. (**E**–**G**) Structures with glycans. The main structure is colored according to the secondary structure: yellow for β-strand, magenta for α-helix, blue for 3–10 helix, and cyan for turns. The cleavage site (Y1605-M1606) is colored in red and the termini are in van der Waals spheres with Cter in orange and Nter in gray. Glycans are colored according to atom types and shown as sticks: O in red, N in blue, C in cyan, and S in yellow.

von Willebrand Factor (vWF) is a blood glycoprotein that binds to platelets and collagen in the process of hemostasis. vWF is known to be synthesized and secreted by vascular endothelial cells and megakaryocytes^1,2^ and has the major function of adhering platelets to the subendothelial extracellular matrices at the broken vessel. At its matured form, a monomeric vWF has a total of 2,050 residues, and its 13 domains are connected in the following sequence: D′-D3-A1-A2-A3-D4-C1-C2-C3-C4-C5-C6-CT/CK (Fig. 1A)^3^. A dimer is formed by two monomers connected by disulfide bonds between CK domains. Dimers are polymerized into large multimers by disulfide bonds connecting D3 domains, making molecular weight up to 20,000 kDa^4^. Specifically, for hemostasis process, the D′ and D3 domains bind to clotting factor VIII^5^, while the A1 domain binds to platelet glycoprotein 1b (GP1b)^6–8^, collagen type VI^9^, and heparin^10^. The A3 domain contains the binding sites for collagen at the damaged vessel spot. Nonetheless, it has been recently noted that the A1 domain could be shielded from binding to platelets due to its N- and C- terminal flanking regions and the adjacent A2 domain^11,12^. The A2 domain can be sufficiently extended or even unfolded under increased hydrodynamic loading, which may permit the A1 domain to interact with platelets. The A2 domain also contains a proteolytic site for a plasma protease (ADAMTS13: A Disintegrin and Metalloprotease with a ThromboSpondin type 1 motif, member 13) to regulate the size of vWF multimers^13^. Various asparagine (Asn) residues have been found to be highly glycosylated, and the number of N-linked glycans carried by typical sized vWF multimer is reported to be more than 300. For the A2 domain only, several glycoforms are determined on two specific sites, N1515 and N1574^14^.

Interestingly, a distinction of blood clotting stages has been characterized when vWF exhibits different types of molecular behaviors^15^ that are observed on two separate scales: whole polymer elongation and individual domain unfolding. For the former, Schneider *et al*. and Ouyang *et al*. independently showed that vWF multimers adopt a globular conformation in quiescent and low extension flow conditions. When extension rate exceeds a threshold value (usually when shear rate is over 1000 s^−1^), vWF multimers transit to an elongated conformation^16,17^. For the individual domain unfolding behavior, A2 domain unfolding at high extension rate is preceded by domain elongation at intermediate extension rate. Experimental studies also demonstrated that ultralarge vWF (ULVWF) multimers, secreted from blood before released from Weibel-Palade bodies in endothelial cells^2,18^, were easier and more efficient to bind to GP1b than plasma vWF^19^. This is because, for a given extension rate, ULVWF multimers exhibit more elongation, exposing more sites for binding, than typical vWF in plasma. In healthy conditions, ADAMTS13 would cleave the peptide bond between Tyr1605 and Met1606 in A2 domains in the ULVWF multimers^20^; this prevents thrombosis and maintains a proper size range for functional vWF in blood. However, the cleavage site is located in the central part of the A2 domain and can be exposed only when the A2 domain is unfolded^2,21,22^. In disease conditions, this size regulation process could be disrupted. For example, clots formed due to aggregation of ULVWF multimers are reported to be one of the causes of thrombotic thrombocytopenic purpura (TTP)^13,23^. A significant number of Type 2 von Willebrand disease (vWD) is caused by mutations of the A2 domain, which may have some relationship with easy exposure of the cleavage site and thus its cleavage, resulting in a deficiency in making sufficiently long vWF to initiate clotting and stop bleeding^24,25^.

Experiments and simulations have been employed to elucidate the mechanical properties of vWF A2 domain unfolding and associated ADAMTS13 cleavage. Near to the wound site on a vessel wall, the extension rate increases dramatically, exerting large extension forces on vWF multimers. These extension forces would likely to invoke some A2 domains into unfolded states, so that the platelet/collagen binding and ADAMTS13 regulation process can be accelerated^26^. To mimic the flow induced unfolding process, external forces can be applied to the terminals of an A2 domain to move them apart. This can be done in experiments by optical tweezer and atomic force microscopy techniques or in atomic scale simulations. Chen *et al*. performed molecular dynamics (MD) simulations based on a published homology model of the A2 domain^27,28^, which gave first insight into force induced A2 domain unfolding sequence. Interlandi *et al*. later also performed force-induced unfolding simulations based on a crystal structure^29^. They concluded that the mutations related to certain vWDs (L1657I, I1628T, and E1638K) made the unfolding of the A2 domain easier than wild-type (WT) under similar external forces. This is indeed consistent with some vWDs related to those mutations whose certain hydrophobic residues were changed into charged side chains. McKinnon *et al*. reported that N-linked glycan itself could interact with ADAMTS13, preventing the A2 domain from being cleaved^30^. This work gave first insight into the N-linked glycans’ influence on ADAMTS13 proteolysis process of A2 domain.

Recent biochemical and biophysical studies elucidated that the vicinal disulfide bonds in the C-terminal of A2 (C1669-C1670) with Ca^2+^ can protect A2 domain cleavage by ADAMTS13^31–33^. An experimental study showed that the N-linked glycan on N1574 also stabilized the A2 domain against ADAMTS13 proteolysis^34^. In this work, Lynch *et al*. have performed experiments with WT A2 and truncated glycan structures for thermal-induced unfolding and the ADAMTS13 proteolysis. They concluded that glycans could essentially prohibit ADAMTS13 binding, suggesting that N-linked glycans can protect the already unfolded A2 domains from being cleaved by ADAMTS13. This result gives more insightful evidence about roles of glycan N1574 in modulating ADAMTS13 proteolysis process. In blood, as described previously, due to the high shearing and extension at the wound spot, the A2 domain undergoes unfolding by flow-induced forces much more than by thermal fluctuation. Thus it is still unclear whether and how N-linked glycans influence the force-induced unfolding process of the A2 domain.

In this study, we performed MD simulations aiming to elucidate the influence of N-linked glycans on force-induced A2 domain unfolding process by comparing unfolding sequence, unfolding force, as well as energy with and without N-linked glycans. The N-linked glycans in this study were chosen to be the glycoforms found in human blood^14^. Moreover, there are also researches showing that vWF proteolysis by ADAMTS13 is not a fast on/off process^26,35^, so that the simulations in this study were performed until the A2 domain was fully extended^36^ to provide insight into its size regulation process. To further investigate the influence of glycans on protein structure, standard (thermal equilibration) MD simulations of A2 domain with and without glycans (each with two replicas) were also performed.

## Results

### N-linked Glycans can change unfolding sequence of A2 domain

As shown in Fig. 1, the A2 cleavage site (Y1605-M1606) by ADAMTS13 is buried at the central part of the A2 domain, which is not accessible to ADAMTS13 or even water molecules when the A2 domain is folded. To explore the influence of two N-linked glycans to the force-induced unfolding process, we performed four different pulling simulations (see Methods for details): ${S}_{N}^{+G}$ (pulling Nter with glycans), ${S}_{C}^{+G}$ (pulling Cter with glycans), ${S}_{N}^{-G}$ (pulling Nter without glycans), and ${S}_{C}^{-G}$ (pulling Cter without glycans).

**Figure 2:**
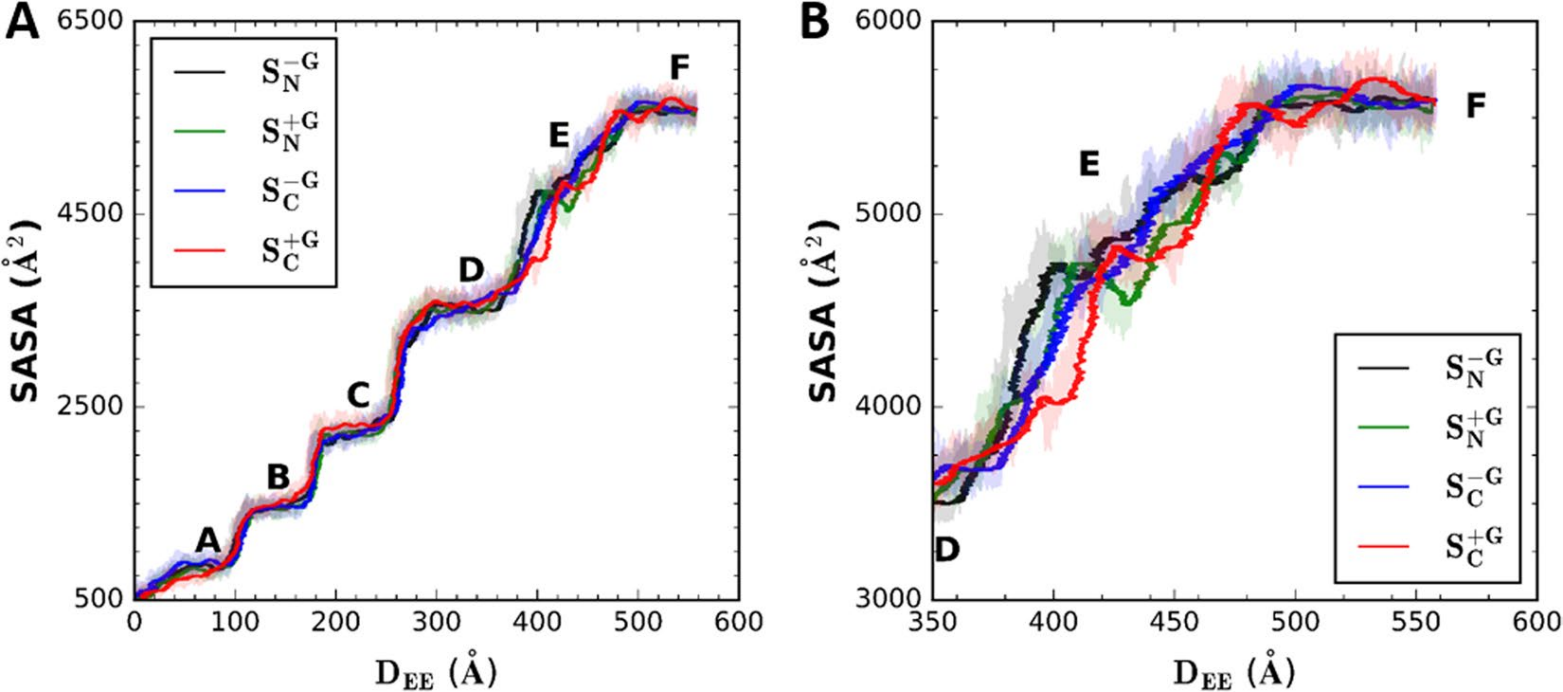
Solvent accessible surface area (SASA) profiles as a function of end-to-end distance, *D*_*EE*_. Using a probe solvent radius of 1.4 Å, the SASA calculations were done only for the β-strands until the *D*_*EE*_ reaches 570 Å, the fully extended length of a single A2 domain. The red line is for the system ${S}_{C}^{+G}$, green for ${S}_{N}^{+G}$, blue for ${S}_{C}^{-G}$, and black for ${S}_{N}^{-G}$. These profiles are obtained by averaging every 1000-step from the raw data (shaded). The SASA profiles are shown for (**A**) entire pulling duration and (**B**) only for states E and F.

Figure 2 shows the solvent accessible surface area (SASA) profiles as a function of terminal-to-terminal distance (i.e., end-to-end distance, *D*_*EE*_). There are six representative SASA states during the unfolding process, and a clear jump in SASA can be seen between two adjacent states, representing an unfolding event of one of the β-strands. These states represent: (A) no β-strand unfolding, (B) β6 unfolding, (C) β5 unfolding, (D) β4 unfolding, (E) β3/β1 unfolding, and (F) fully unfolded A2, respectively. More specifically, state A represents the state where each β-strand is in a folded state, and only α6 is pulled out from the main body of the A2 domain. Thus, the sudden SASA jump between states A and B represents β6 unfolding. The same trend goes as each β-strand unfolds. And, as shown by Chen *et al*.^27^, pullouts of β5 or β4 is a sliding pathway which shows a clearer SASA jump. On the other hand, β1 unfolding is more like an unzipping pathway, going with a gradual SASA increase shown between states D and E. Clearly, the unfolding of either β6, β5, or β4 among all four systems happens almost at the same *D*_*EE*_, showing overall significant overlaps in the different pulling schemes. However, for the transition from state D to E (Fig. 2B), the SASA curves of these four systems exhibit some notable differences, and there is a shift for the ${S}_{C}^{+G}$ system. The reason for this shift is that, unlike the other three systems, the ${S}_{C}^{+G}$ system has β3 unfolded before β1. The competition between β3 and β1 for unfolding makes a delay to achieve state E in ${S}_{C}^{+G}$. This is part of the influence of N-linked glycans on the force-induced unfolding process, which is discussed in detail below. After all the β-strands are unfolded, the SASA of all systems converge to the same value, *i*.*e*., the SASA of the fully extended A2 domain.

**Figure 3:**
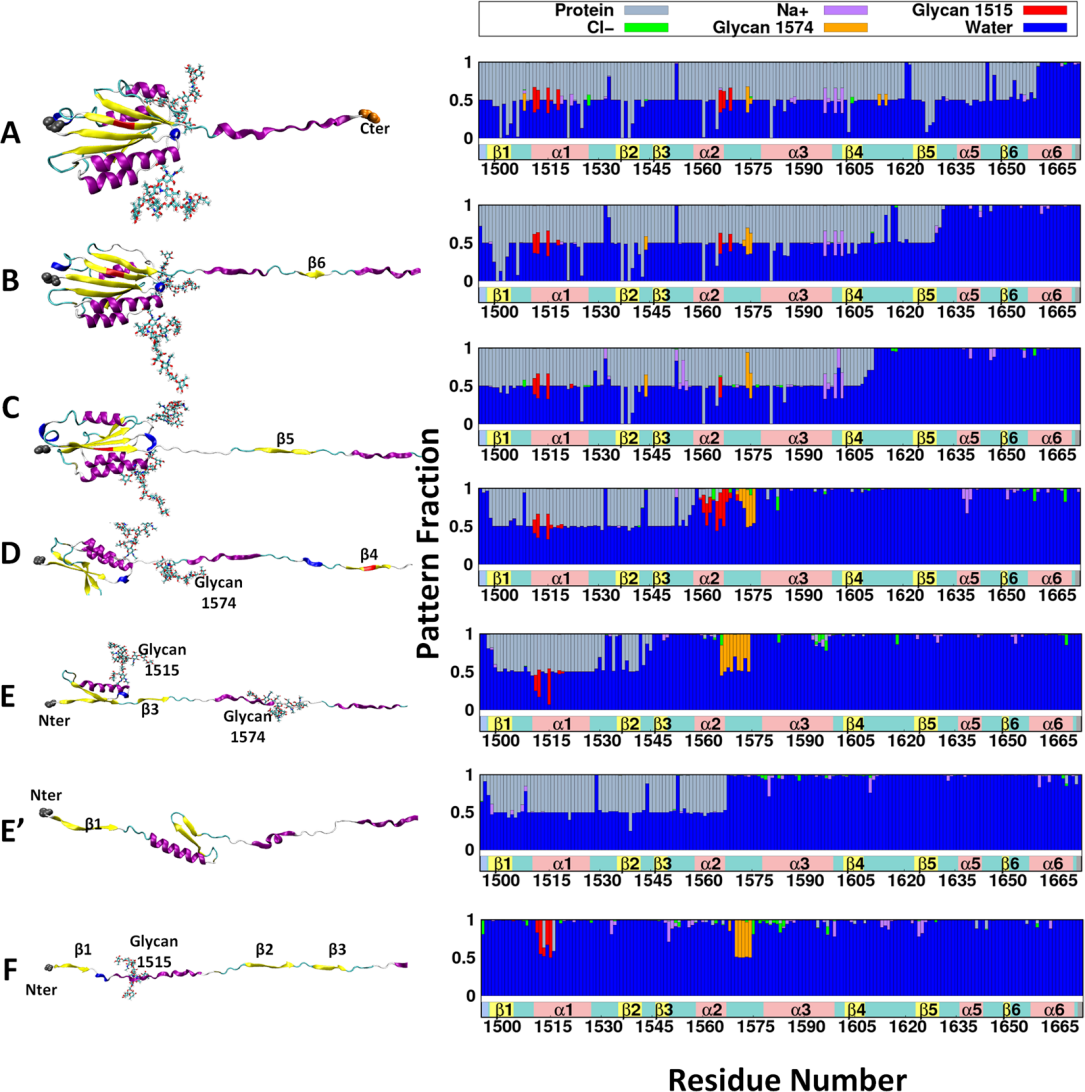
A2 Domain Unfolding Sequence. The left panel shows the structure of each unfolded state in Fig. 2. The right panel shows the averaged interaction pattern of each protein residue with its environment over the certain state. The interaction pattern graph shows the frequency of occurrence within 4 Å from each of other protein residues (gray), water molecules (blue), Cl^−^ (green), Na^+^ (purple), glycan N1515 (red), and glycan N1574 (orange). The structure has been colored in the same way as in Fig. 1, and labels are shown only for the unfolded structures. States (**A**–**F**) represent ${S}_{C}^{+G}$, while state E′ shows ${S}_{C}^{-G}$ for different unfolding sequence at state E(/E′).

Figure 3 shows a representative conformation of each unfolded state (same as in Fig. 2), and the interaction pattern of each residue in the A2 domain. Unfolding happened gradually following the sequence of β6, β5, β4, β1/β3. When the corresponding structure unfolds (e.g., comparing state A with B for β6), the frequency of having water molecule within a distance of 4 Å from β6 residues increases dramatically from 50% to almost 100%. This indicates that, before β6 unfolds, its one face is fully involved in hydrogen bonds (H-bonds) with β5 and can rarely interact with water molecules; after unfolding, both faces of β6 are accessible to the solvent, thus the interaction frequency with water molecules increased to 100%. β6 unfolding can also be seen in the decreased protein (or increased water) interaction frequency for β5 in going from state A to B. Before β6 unfolds, β5 spends around 80% of time interacting with the rest of the protein residues and only 20% with water molecules (including ions). After β6 unfolds, however, these values change to be half and half, meaning that one face of β5 becomes exposed to solvent and the other one is still involved in the H-bonds with the rest of the protein (β4 in this case).

Interestingly, there are some long-range protein-glycan interactions shown in Fig. 3. The glycan structure is relatively large, e.g., 28.54 Å for glycan N1515 and 27.95 Å for glycan N1574 in length along the longest axis of the glycan structure, which can be compared with 38.58 Å that is the folded A2 backbone length along its longest axis. Thus, it is possible that these N-linked glycans can not only interact with the residues to whom they are attached or near neighbors, but also have some long-range interactions with residues that are relatively farther away. For instance, before these glycan-bearing residues unfolded, glycan N1574 interacts with residues K1508 (6.2 Å), S1542 (10.3 Å), S1613 (9.1 Å), and E1615 (16.0 Å), where each distance measured between glycan bearing residue and the corresponding residue. Glycan N1515 also shows long-range and long-lasting interactions with α2 helix: residues D1560 (18.4 Å) and Q1571 (11.8 Å) before α2 is pulled out. These long-range protein-glycan interactions are not rare. For more than 72% of the time (i.e., during 83 ns of 114 ns), at least one glycan shows interactions with distal residues besides its near neighbors (see also the Supplemental Movie S1 for interaction pattern changes as a function of time).

For states E and E′, as shown in Fig. 3, the unfolding sequence, whether β1 unfolds before β3 or *vice versa*, shows a strong relationship with the long-range interactions between glycan N1515 and α2 helix. A typical unfolding sequence without glycans^27^ is shown in state E′, i.e., β1 unfolds before β3, and then the whole domain reaches the full extension. As shown in Fig. 3E′, after β1 is pulled out, α2 still maintains a high protein-protein interaction frequency; this persists until H-bonds between β3 and β2 are broken. This indicates that the energy needed to break H-bonds between β3 and β2 may not be larger than the energy needed to unfold α2. Unlike the sliding pathway of β4/β5 that requires H-bonds to break all at once, β1/β3 unfolding undergoes an unzipping pathway. This allows H-bonds to be broken gradually with a moderate energy requirement, indicating that H-bonds β2 forms with either β1 or β3 are relatively weak.

**Figure 4:**
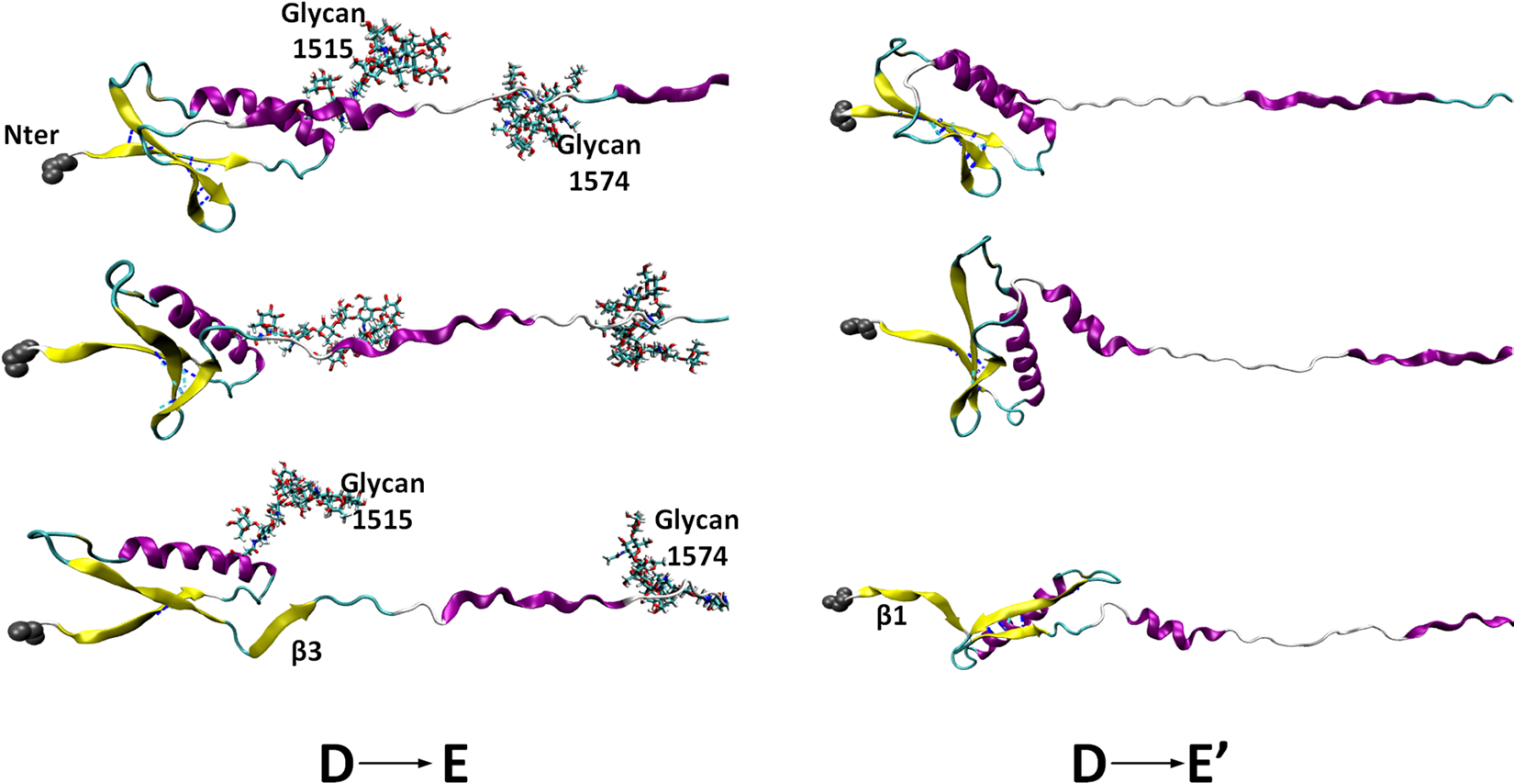
The left panel shows the snapshots for the unfolding sequence of system ${S}_{C}^{+G}$ with β3 pulled out before β1, and the right panel shows the opposite unfolding sequence of system ${S}_{C}^{-G}$. Both panels show the unfolded states from D to E(/E′) (*D*_*EE*_ = 420 to 540 Å): the top panels for state D, bottom panels for state E (/E′), and the middle panels for an intermediate state.

As clearly shown in Fig. 4, within the same range of *D*_*EE*_, the unfolding sequence for system ${S}_{C}^{+G}$ (state D to E) is different from system ${S}_{C}^{-G}$ (state D to E′). While glycan N1515 interacts with α2 helix (Fig. 3C), due to glycan N1515’s location near the end of β1, β1 also stays close to α2. When this interaction ends, the folded structure containing glycan N1515 moves away from α2, exposing β3, which is then pulled out. It is still interesting to point out that when β3 is pulled out, β1 is almost half-way departed from β2 and the count of H-bonds in this state is about 50% less than that in state D. Even though the unfolding sequence has been changed due to the glycan N1515 and α2 helix interaction, the opposite unfolding sequence may still be possible for system ${S}_{C}^{+G}$. If one compares the transition from state D to E with that from state D to E′, before any β-strand fully unfolds, there is an intermediate state where β2 has less than half count of fully folded H-bonds with either β1 or β3 (Fig. 4 middle panel). In this state, whether β3 or β1 unfolds first may easily be influenced by some instantaneous conditions, e.g., details of the protein-glycan interaction at that conformation. Supplementary Information Fig. S1 describes the results of an additional pulling simulation (${S}_{C(repeat)}^{+G}$) with glycans from state D, indicating that the protein-glycan interactions are stochastic (at least during our simulation time) and β1 can unfold before β3 even with protein-glycan interactions. From Fig. S1A, it is clear that system ${S}_{C(repeat)}^{+G}$ shows more overlapping with other systems than system ${S}_{C}^{+G}$. This is because β1 unfolds before β3 in ${S}_{C(repeat)}^{+G}$. This similar unfolding sequence as in systems ${S}_{C/N}^{-G}$ gives the SASA jump at similar *D*_*EE*_. Nonetheless, system ${S}_{C(repeat)}^{+G}$ (Fig. S1C) also clearly show that, when β1 unfolds, β3 is almost ‘half-unfolding’. Comparing with Fig. 4, it indicates that under the protein-glycan interaction, no matter that β1 unfolds before β3 or *vice versa*, when one β-strand unfolds, the other one is about half-departed, which has not been seen in the systems ${S}_{C/N}^{-G}$. Moreover, the unfolding energetics (see below for details) is not much different between systems ${S}_{C(repeat)}^{+G}$ and ${S}_{C}^{+G}$ (Fig. S1B,D). This indicates that the unfolding sequence of β1-β2-β3 is relatively stochastic. Whether the unfolding sequence depend on different glycoforms, and how this unfolding sequence influences on vWF functionality are interesting questions that require further investigations.

### N-linked Glycans stabilize A2 domain from force-induced unfolding

**Figure 5:**
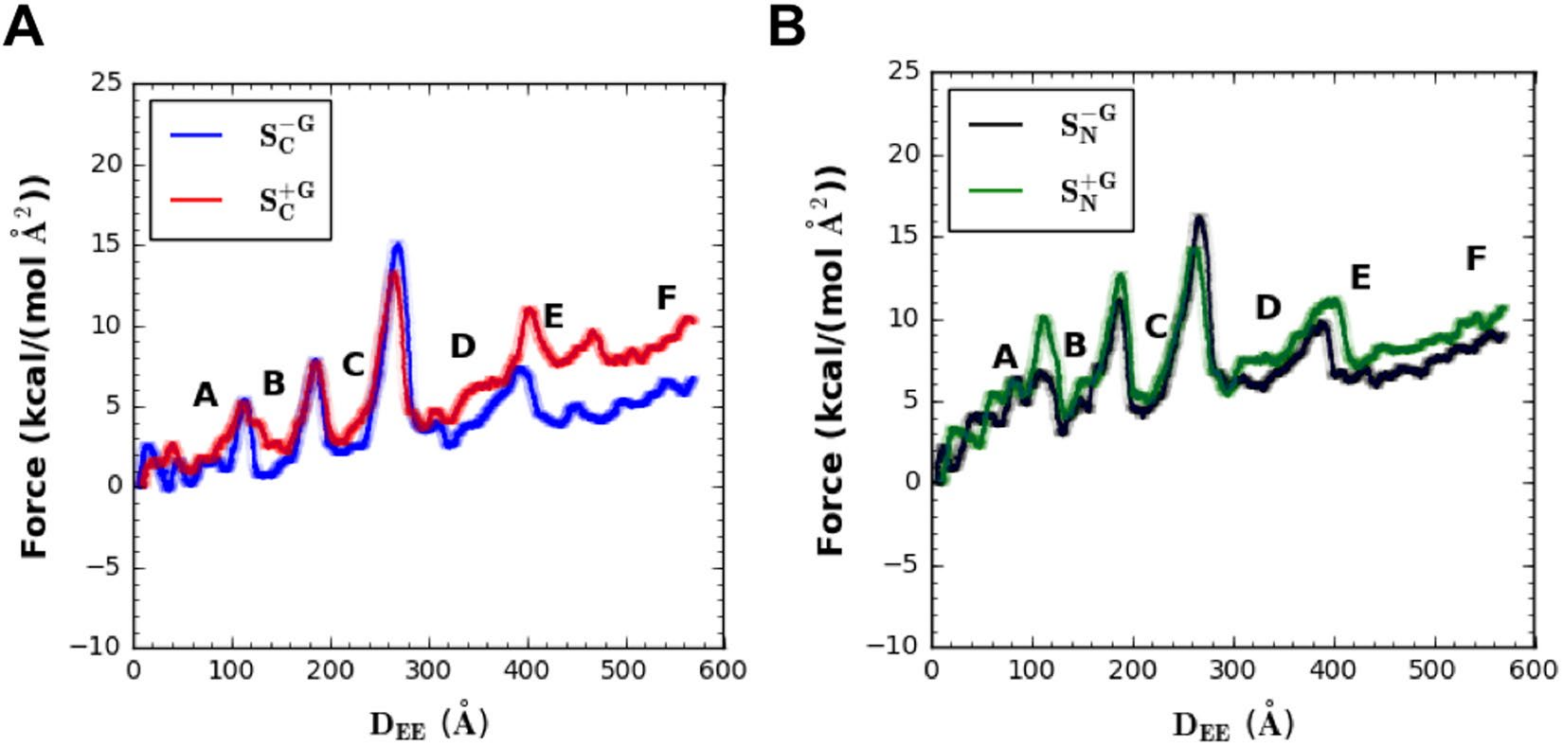
Force profiles as a function of *D*_*EE*_ for different systems, (**A**) ${S}_{C}^{+G}$ (red) and ${S}_{C}^{-G}$ (blue) and (**B**) ${S}_{N}^{+G}$ (green) and ${S}_{N}^{-G}$ (black). Different states have been labeled according to Fig. 2. Solid lines are the averages over every 1000-step of the raw data, and the shaded lines represent the 95% confidence intervals^56^. Note that 1 kcal/(mol·Å^2^) = 69.7 pN.

The long-ranged protein-glycan interactions not only influence the unfolding sequence, but also increase the instantaneous forces and energy for A2 domain unfolding. The instantaneous force profiles as a function of *D*_*EE*_ are shown in Fig. 5. Consistent with Fig. 2, each of the main peaks in the force profile matches an unfolding event of a single β-strand. Forces were calculated based on the distance between the dummy point carrying the spring potential and the terminal center of mass (COM). Note that forces become negative values when a certain terminal COM moves more than a target position (defined by the dummy point) due to the thermal fluctuation. This could be considered to be evidence that the pulling speed in this work is low enough to probe a realistic response (although a pulling speed of 500 mm/s in this study is still much higher than those used in experiments whose usual highest pulling speed reaches 0.01 mm/s in atomic force microscopy^36,37^, or at most 0.1 mm/s^38^).

Force fluctuations can be seen before β6 unfolds due to pre-adjustments of protein conformations during early pulling stages. In fact, the largest differences in the force profiles for pulling Nter versus Cter occur during the first few unfolding events (i.e., before β4). This is because β6 (close to the Cter) is always the first β-strand that is pulled out regardless of the Nter or Cter pulling. For the Cter pulling, residues close to the Cter are pulled out from the rest of the A2 domain that remains relatively stationary. For the Nter pulling, instead of pulling out the residues close to Nter, the bulk of the A2 domain is re-oriented and moves with the Nter group. Thus, in addition to the forces/energies required to pull out residues close to the Cter, additional forces/energies are required to move the bulk of the A2 domain with the Nter group. So that, the external forces can be transmitted to the structure near the Cter, overcoming the energy barrier to break the H-bonds for β6 unfolding (Fig. 3 states A and B).

In order to investigate whether such pre-adjustments have influences on the overall unfolding evens, we performed another independent simulation for each pulling Cter/Nter strategy up to β6 or β5 unfolding (${S}_{C(replica)}^{+G}$ and ${S}_{N(replica)}^{+G}$ with glycans and ${S}_{C(replica)}^{-G}$ and ${S}_{N(replica)}^{-G}$ without glycans) and the results are shown in Supporting Information Fig. S2. From Fig. S2A, it is clear that the unfolding evens also happened at the same *D*_*EE*_ (comparing β6 or even β5 unfolds). Moreover, Fig. S2B–D show that there is little difference for forces/energy profiles when comparing with independent simulations (see below for energy profile discussion in detail). These results confirm that, even though such pre-adjustment processes are somewhat stochastic, there is not much influence on the overall unfolding events.

**Figure 6:**
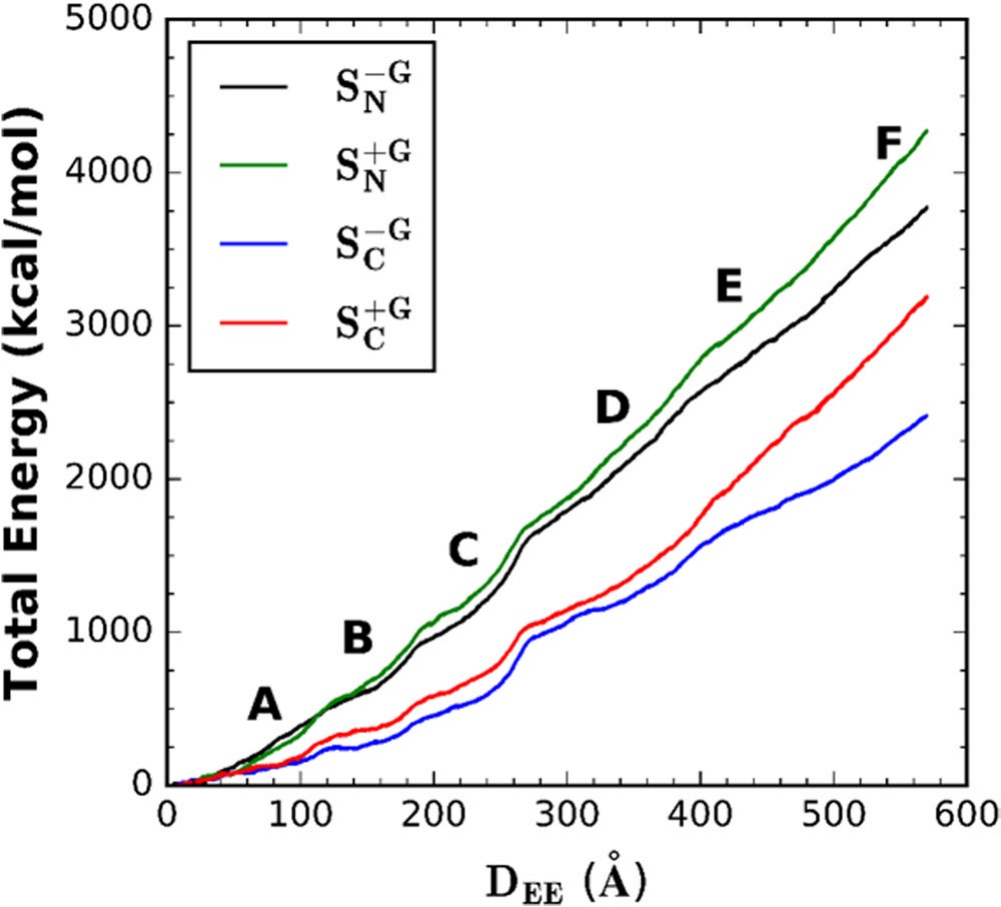
Energy required to unfold A2 as a function of *D*_*EE*_ for different systems: ${S}_{C}^{+G}$ (red), ${S}_{C}^{-G}$ (blue), ${S}_{N}^{+G}$ (green), and ${S}_{N}^{-G}$ (black). Different states are labeled according to Fig. 2. Note that 1 kcal/mol = 69.7 pN·Å.

Regardless of which terminal group is pulled, when *D*_*EE*_ exceeds 300 Å (i.e., during the second half of domain unfolding), the difference between force profiles with glycans versus without glycans starts to increase (Fig. 5). A clear force separation can be seen between the lower bound of ${S}_{C/N}^{+G}$ and the upper bound of ${S}_{C/N}^{-G}$. This separation suggests a statistically significant difference between systems ${S}^{+/-G}$ over a wide range (i.e., after β4 unfolds) independent of pulling strategy. In addition, the cumulative effect of forces (i.e., energy) also justified the observed differences (Fig. 6, which will be elaborated below). As mentioned previously, β4/β5 unfolding undergoes a sliding pathway, so that more forces are required to break all the H-bonds at once. Thus, protein-glycan interactions may not show much influence on instantaneous force profiles of β4/β5 unfolding event. However, other β-strands’ unzipping unfolding (i.e., after β4 unfolds) that requires relatively moderate forces can be easily influenced by protein-glycan interactions.

Figure 6 shows the energy profiles for A2 domain unfolding as a function of *D*_*EE*_. The systems with glycans (${S}_{C/N}^{+G}$) require more energy (around 23% more) than the systems without glycans (${S}_{C/N}^{-G}$) for full unfolding. This energy also varies depending on different terminals to be pulled. Within the same terminal pulling strategy, the energy ratios between the systems with or without glycans are 1.32 (${S}_{C}^{+G}$/${S}_{C}^{-G}$) and 1.14 (${S}_{N}^{+G}$/${S}_{N}^{-G}$) when *D*_*EE*_ = 570 Å. It is also important to note that the energy profile here does not show a linear increase with *D*_*EE*_. Instead, there exist some small sudden increases. Each of these small jumps happens at similar *D*_*EE*_ among all systems. When compared with Figs 2 and 5, each *D*_*EE*_ for small jumps corresponds to the value where each β-strand is pulled out. This indicates that the β-strand unfolding process needs to overcome the energy barriers associated with breaking H-bonds, and the energy increases in a more moderate way to extend the unfolded structure after each β-strand is pulled out.

The energies required to fully extend the A2 domain for systems ${S}_{N}^{+/-G}$ are higher than those for systems ${S}_{C}^{+/-G}$. This is even true when comparing ${S}_{N}^{-G}$ with ${S}_{C}^{+G}$. As mentioned above, this is due to the fact that β6 (close to the Cter) is always the first β-strand that is pulled out even in systems ${S}_{N}^{+/-G}$ where the Cter is fixed. It is also interesting to point out that even only considering β6-β5-β4 unfolding events, the cumulative energy is still higher (about 10% more) for systems with glycans compared to those without. Thus, even if considering only up to the point when the cleavage site (which is reported to be on β4) becomes exposed, results here still give evidence that the total energy required for unfolding in systems ${S}_{C/N}^{+G}$ is higher than ${S}_{C/N}^{-G}$. So that N-linked glycans stabilize force-induced unfolding of A2 domain regardless of unfolding to a fully extended form or until the cleavage site becomes exposed.

To examine the influence of the viscous force (due to the bulky glycans) on the force difference between systems ${S}_{C/N}^{+G}$ and ${S}_{C/N}^{-G}$ in Fig. 5, four independent pulling simulations were performed for systems ${S}_{N}^{+G}$ and ${S}_{N}^{-G}$ without fixing any atom. These independent pulling simulations are named ${S}_{N(add)}^{+G}$ and ${S}_{N(add)}^{-G}$. The spring constant and moving speed in ${S}_{N(add)}^{+/-G}$ is the same as those in systems ${S}_{N}^{+/-G}$. Figure S3 shows the resulting force/COM profiles in systems ${S}_{N(add)}^{+G}$ and ${S}_{N(add)}^{-G}$. As shown in Fig. S3A, the forces in both systems begin with an increase and gradually fluctuate around 0.5 kcal/(mol Å^2^). ${S}_{N(add)}^{+G}$ show a slightly higher force initially and larger fluctuations compared to ${S}_{N(add)}^{-G}$ due to additional mass from bulky glycans. When averaging over four independent simulations, both systems show a relatively stable force profile after 5 ns. In addition, the COM profiles (Fig. S3B) become parallel after 5 ns, indicating an equilibration over this period. Thus, 10 ns simulation is sufficient to demonstrate the glycans’ influence on the viscous force. The calculated average force difference between ${S}_{N/N(add)}^{+G}$ and ${S}_{N/N(add)}^{-G}$ is 0.026 kcal/(mol Å^2^), which is slightly over 2% of that in ${S}_{N}^{+/-G}$ (i.e., 1.117 kcal/(mol Å^2^)). Clearly, the viscous force contribution to the force difference in Fig. 5 is negligible. Therefore, we conclude that the force difference arises from protein-glycans interactions rather than the bulky glycan viscous effects.

### N-linked Glycans do not change A2 domain structure

**Figure 7:**
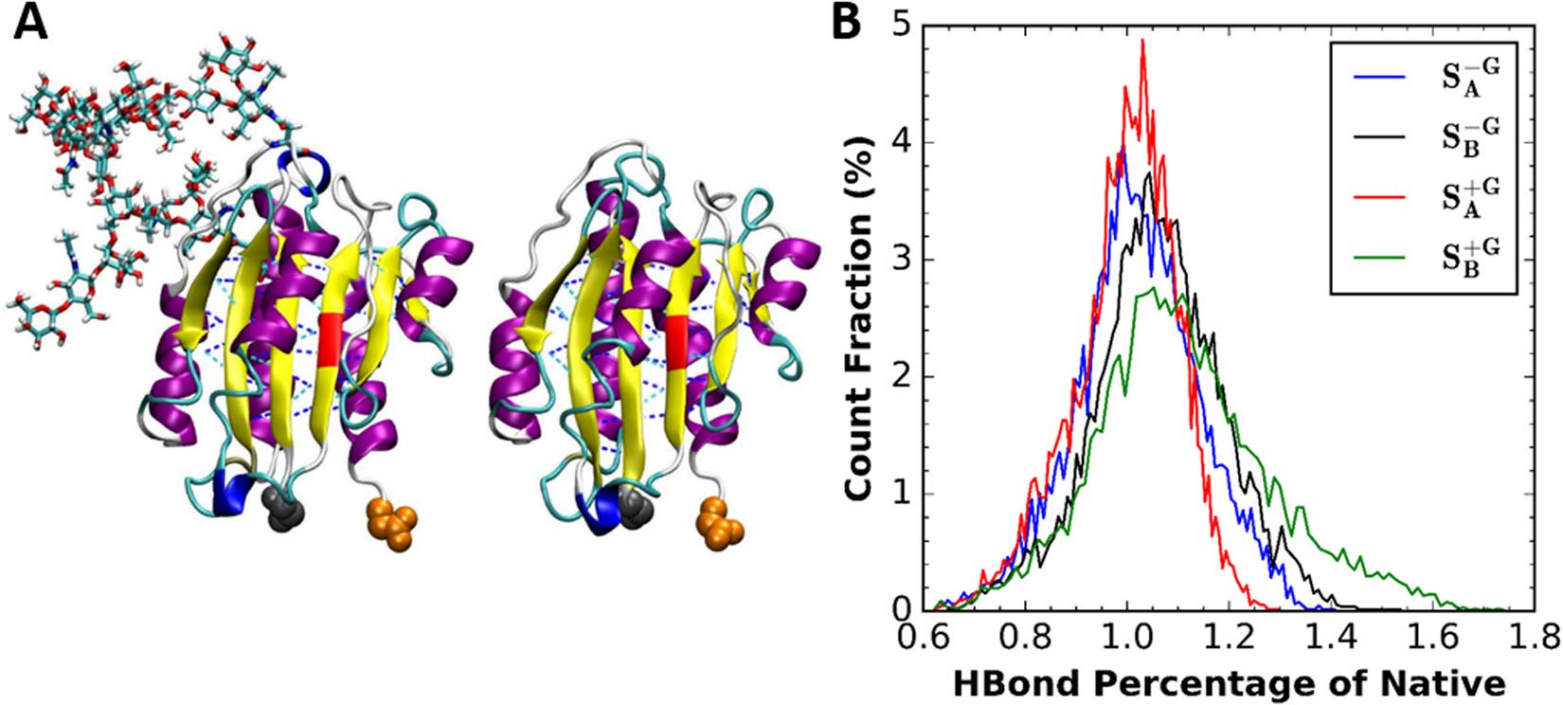
(**A**) Snapshots from standard MD simulations with/without glycans. (**B**) Histograms of the ratio of the number of H-bonds among β-strands in simulations to that in the crystal structure: ${S}_{A}^{+G}$ (red), ${S}_{B}^{+G}$ (green), ${S}_{A}^{-G}$ (blue), and ${S}_{B}^{-G}$ (black). Note that H-bonds were defined with a cut-off distance of 3.5 Å and the angle between donor, hydrogen, and acceptor residues greater than 120°.

To investigate the reason why systems ${S}_{C/N}^{+G}$ require higher unfolding forces/energy than systems ${S}_{C/N}^{-G}$, standard (thermal equilibration) MD simulations with or without glycans each with two different replicas (${S}_{A/B}^{+G}$ and ${S}_{A/B}^{-G}$, respectively) were performed. These simulations used the same initial structure as those in pulling ones and ran for 500 ns without any external force. H-bonds were counted only among β-strands. Figure 7 shows the histograms of the ratios of the H-bond count during the simulation with respect to that in the crystal structures. Clearly, the number of H-bonds does not show much difference among the systems: the H-bond ratios are 1.05 ± 0.25 (${S}_{A}^{+G}$), 1.10 ± 0.65 (${S}_{B}^{+G}$), 1.02 ± 0.40 (${S}_{A}^{-G}$), and 1.07 ± 0.63 (${S}_{B}^{-G}$). This means that N-glycans do not change H-bond patterns between β-strands. Thus, there is barely any structural change that makes systems ${S}_{C/N}^{+G}$ harder to unfold when the A2 domain is properly folded. On the other hand, Figs 3 and 4 show clear protein-glycan interactions. Therefore, the long-ranged protein-glycan interaction prohibits part of protein from unfolding, which makes the unfolding force/energy higher than systems ${S}_{C/N}^{-G}$.

## Discussion

It has been shown that N-linked glycans of vWF A2 domain can prohibit its binding with ADAMTS13 and thus proteolysis by ADAMTS13^34^. Here, we present a mechanistic study focusing on the influence of N-linked glycans on the force-induced unfolding of the A2 domain. We have performed MD simulations of force-induced unfolding process with the crystal structure of vWF A2 domain. Glycans were built based on two natural glycoforms^14^. Force-induced pulling was achieved with strategies of pulling through different terminals. Comparing among four independent pulling simulations, the glycans show some influences on the unfolding sequence and required forces/energy. Due to the long-ranged protein-glycan interactions on certain residues, β3 was pulled out earlier than β1 in some glycan systems while the opposite sequence happened in the systems without glycans. In addition, the systems with glycans showed about 25% more energy for full extension and 10% more until exposure of ADAMTS13 cleavage site than the systems without glycans. By examining the H-bond count from standard (thermal equilibration) simulations and calculating the interaction patterns among pulling simulations, it is clear that glycans make force-induced unfolding harder not by changing the protein structure in folded states, but through protein-glycan interactions in unfolded states. As mentioned in Methods, the glycan sequence used in this work contains the shared structure occurred in most glycoforms at each site. Thus, our results suggest that interactions between human plasma vWF and glycans are prevalent and influence the unfolding of A2 domain. Glycoforms may vary among individuals with vWDs, which has not been fully understood yet. Nonetheless, regardless of higher levels of brunches or truncated glycan structures, our work provides insight into a general role of glycans in A2 domain unfolding. We hope that our results may contribute to some investigations for glycan-related vWDs.

## Methods

In this work, all MD simulations were performed with NAMD^39^. We used the CHARMM36m force field for protein^40^ and the CHARMM36 force field for carbohydrates^41–43^. A crystal structure for vWF A2 domain (PDB ID: 3GXB) was taken from Protein Data Bank^44^. We used a TIP3P water model^45^ and counter ions of Na^+^ and Cl^−^ with the concentration of 0.15 M were added to neutralize the system. All simulation systems and parameters were set up using CHARMM-GUI^46,47^. Visualization and analysis were done by VMD^48^.

For A2 domain pulling simulations, the protein structure was initially placed in a rectangular water box with a size of 800 Å by 80 Å by 80 Å. The pulling forces were applied to the center of mass (COM) of each terminal residue (i.e., residue number N1495 or C1672). We performed four simulations: pulling N1495 (by fixing C1672) or C1672 (by fixing N1495) with or without glycans at N1515 and N1574. For convenience, pulling N1495 is called “Nter” pulling and pulling C1672 “Cter” pulling. In addition, the following symbols are used for the pulling systems: ${S}_{N}^{+G}$ (Nter pulling system with glycans), ${S}_{C}^{+G}$ (Cter pulling with glycans), ${S}_{N}^{-G}$ (Nter pulling system without glycans), and ${S}_{C}^{-G}$ (Cter pulling without glycans).

The spring constant for pulling was set to be 5 kcal/mol/Å^2^ and its moving speed to be 5 Å/ns. As a previous work showed, the fully extended length of a single A2 domain is 570 Å^22^, thus all simulations stopped when the terminal to terminal (or end-to-end, *D*_*EE*_) distance reached this value, which is approximately 114 ns. The human vWF A2 domain has six β-strands (β1–β6) connected by loops with various lengths^49^, and these strands were used as references for A2 domain unfolding events. In this work, the sequences of glycan N1515 and glycan N1574 (Fig. 1B,C) were chosen according to the previous experimental work^14^. The shortest sequences that are shared among most abundant glycan populations on each site were employed here^14^. Therefore, the glycan sequences used in this work are in majority of human plasma vWF A2 domains. We used the *Glycan Reader & Modele*r module in CHARMM-GUI (http://www.charmm-gui.org/input/glycan) to model both N-glycan structures^50^; *Glycan Reader & Modele*r uses the PDB glycan structures from GFDB (http://www.glycanstructure.org/fragment-db)^51^. Figure 1 also shows atomic scale models of human vWF A2 domain structures with (Fig. 1E,F) or without two glycans (Fig. 1D).

The van der Waals interactions were smoothly switched off over 10–12 Å by a force-based switching function^52^ and the electrostatic interactions were calculated by particle-mesh Ewald method^53^ with a mesh size of ~1 Å for fast Fourier transformation and sixth order B-spline interpolation. SHAKE algorithm was used to constrain bond lengths involving hydrogen atoms^54^ and the simulation time-step was 2 fs. We first relaxed the system in a canonical ensemble (NVT) with T = 300 K with harmonic restraints to all solute atoms. The constant temperature was controlled by Langevin dynamics with the damping frequency of 50 fs^−1^. 100–120 ps isothermal isobaric ensemble (NPT) was then applied to adjust the solvent density. To control the constant pressure, Langevin piston method was used. A dihedral restraint force constant was set to be 1 kcal/(mol·rad^2^) in order to keep the carbohydate chair conformation during these equilibration steps. To perform the force-induced unfolding simulation of A2 domain (i.e., pulling simulations), COLVARS method was used^55^ and the COMs of two terminal residues were calculated firstly as the external forces’ initial positions. The effective spring potential (negative derivative of which is used to represent external forces) acting on a pulling terminal residue was calculated using the following equation:

1 $$U(\overrightarrow{{r}_{1}},\,\overrightarrow{{r}_{2}},\,\overrightarrow{{r}_{3}},\,\mathrm{...},\,t)=\frac{1}{2}k{[vt-\overrightarrow{R}(t)\cdot \overrightarrow{n}]}^{2}$$

where *k* is the spring constant, *v* is the moving speed of the spring potential, $\overrightarrow{R}(t)$ is the current position vector of the terminal COM, and $\overrightarrow{n}$ is the unit vector in the direction along a vector between Nter and Cter COMs. As a result of this spring potential, the spring-connected terminal would move following the energy well, so that part of the A2 domain would be pulled out.

## Electronic supplementary material

Supplementary Information

Movie S1
