# Supplementary material for: Long-ranged Protein-glycan Interactions Stabilize von Willebrand Factor A2 Domain from Mechanical Unfolding
Source: Sci Rep. 2018 Oct 30;8:16017. doi: 10.1038/s41598-018-34374-y (PMC6207679; doi:10.1038/s41598-018-34374-y)
Supplement: Supplementary file 1 — Supplementary Information [file 41598_2018_34374_MOESM1_ESM.pdf]

Supplementary Information

Long-ranged Protein-glycan Interactions Stabilize von Willebrand Factor A2 Domain from Mechanical Unfolding

Chuqiao Dong[1], Jumin Lee[2], Seonghoon Kim[2], Whitney Lai[3], Edmund B. Webb III[1], Alparslan Oztekin[1], X. Frank Zhang[1, 3], and Wonpil Im[2, 3,*]

[1] Department of Mechanical Engineering and Mechanics, Lehigh University, Bethlehem, PA, 18015, United States

[2] Department of Biological Sciences, Lehigh University, Bethlehem, PA, 18015, United States

[3] Department of Bioengineering, Lehigh University, Bethlehem, PA, 18015, United States

Figure S1. (A): SASA profile as a function of D_{EE} for system $S_{C(repeat)}^{+G}$ and those in **Figure 2A**. (B): Energy profile as a function of D_{EE} for systems $S_{C(repeat)}^{+G}$ and S_C^{+G}. (C): Snapshot of structures in state E for systems $S_{C(repeat)}^{+G}$ and S_C^{+G}. Only the unfolded structures are labeled. (D): Force profile as a function of D_{EE} for systems $S_{C(repeat)}^{+G}$ and S_C^{+G}. Solid lines are the averages over every 1000-step of the raw data (shaded lines of the same color).

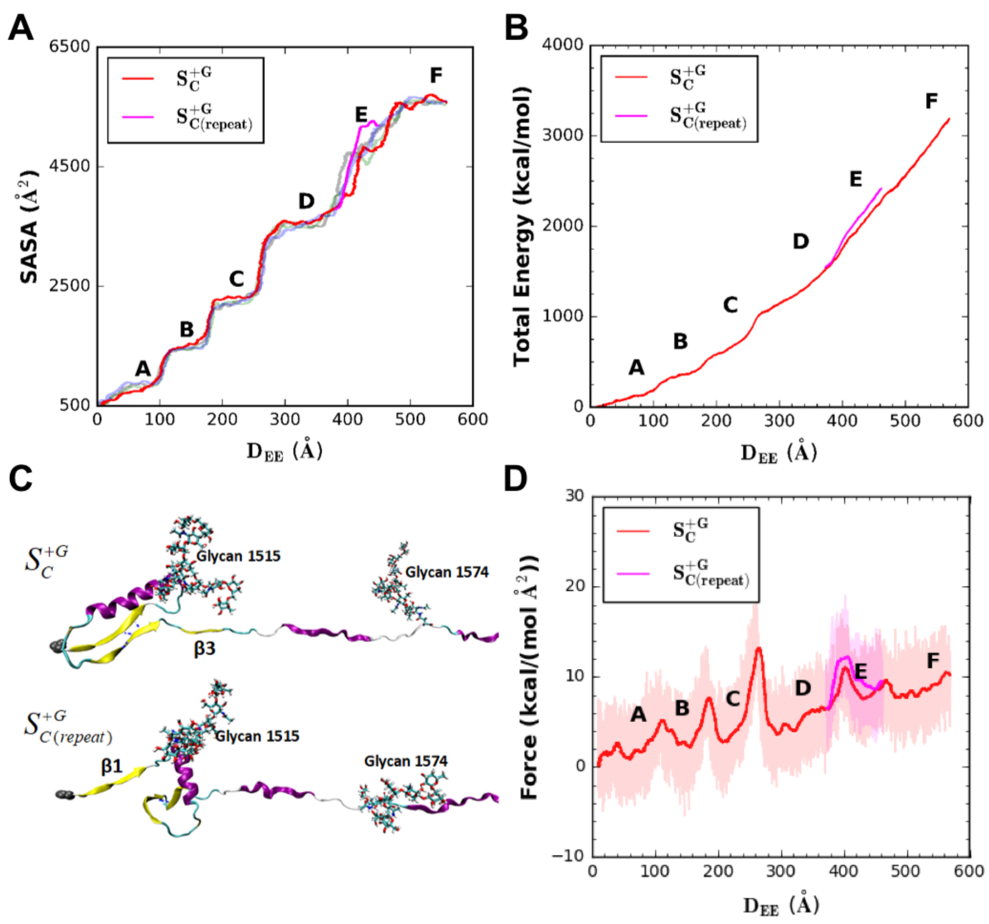

Figure S2. (A): SASA profile as a function of D_{EE} for independent simulations and those in **Figure 2A**. (B): Energy profile as a function of D_{EE} for independent simulations and those in **Figure 6**. (C,D): Force profiles as a function of D_{EE} for independent simulations and those in **Figure 5**. Solid lines are the averages over every 1000-step of the raw data (shaded lines of the same color).

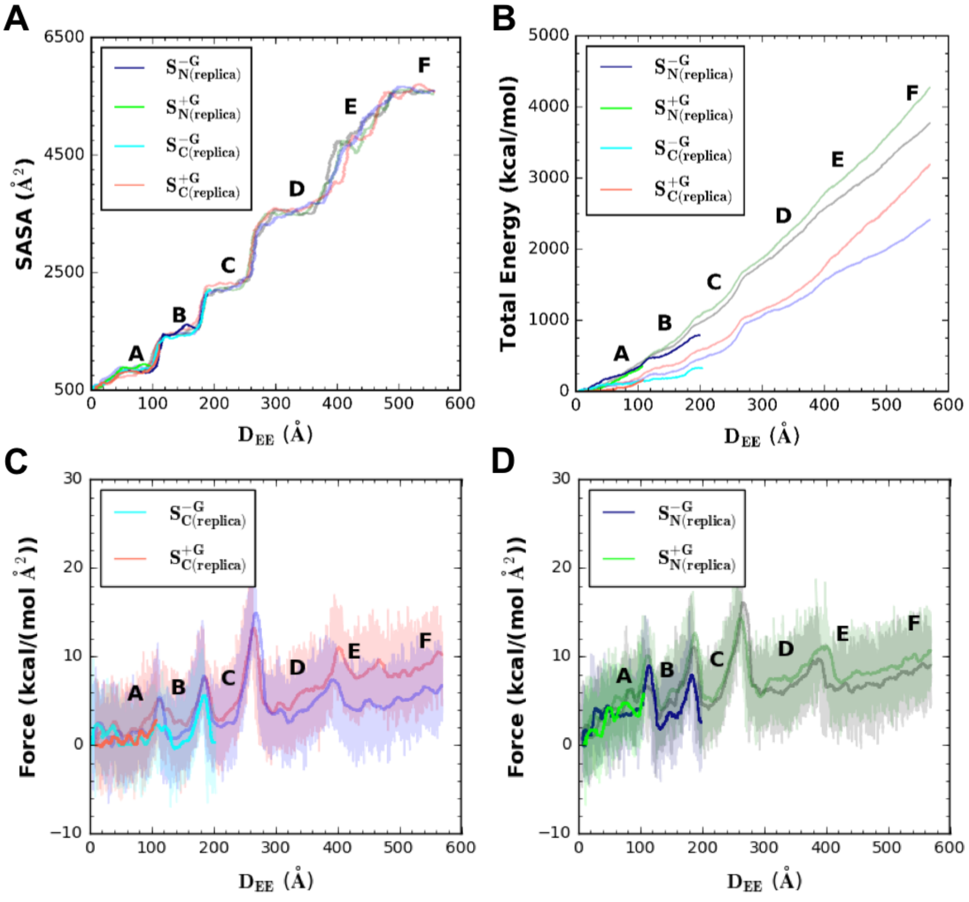

Figure S3. (A) Force profiles for different systems: $S_{N(add)}^{+G}$ (green) and $S_{N(add)}^{-G}$ (back). (B) The corresponding COM profiles. Each solid line represents the averaged value of four independent simulations, and the error bars with the same color indicate the standard errors among them.

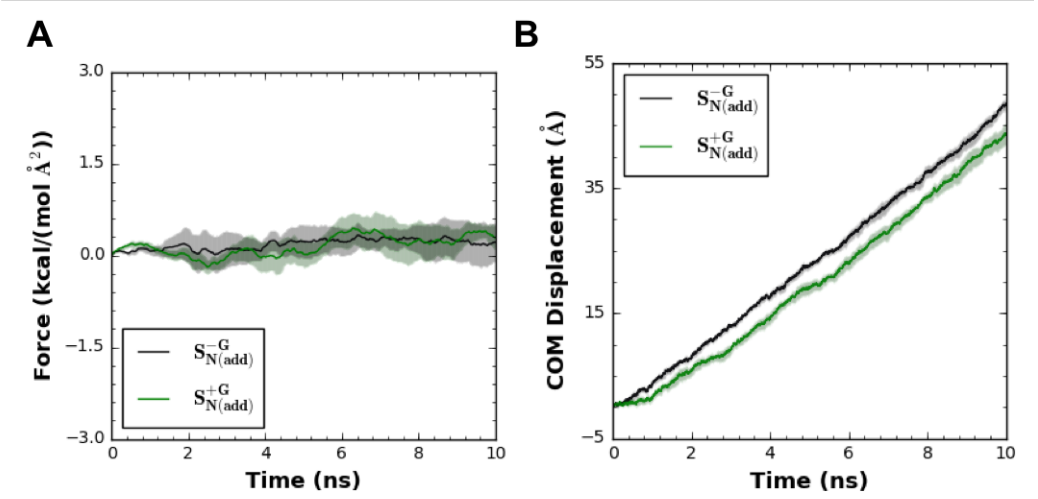

Movie S1. Interaction pattern changes as a function of time.
